# A rare case of post infectious radial hemimelia of right forearm

**DOI:** 10.11604/pamj.2022.42.128.35766

**Published:** 2022-06-16

**Authors:** Devank Lohiya, Parth Shah

**Affiliations:** 1 Department of Orthopedics, Datta Meghe Institute of Medical Sciences, Wardha, Maharashtra, India

**Keywords:** Radial hemimelia, osteotomy, perinatal infection, 3D-computed tomography

## Image in medicine

A problem in the development of components that make up the radial half of the forearm is known as radial longitudinal deficiency (RLD). Radial hemimelia, radial dysplasia, radial meromelia, and radial club hand are all words used to describe the condition. It's an uncommon condition that affects between 1/30,000 and 1/100,000 live births. In nearly half of the instances, there is bilateral involvement. We describe an atypical instance of radial hemimelia in which the radius was significantly hypoplastic while the thumb and carpal bones were normal in size, form, and joint connections. A 16-year-old female presented to orthopedics out patient department (OPD) with complaints of deformity since birth and tingling sensation of right upper limb for 1 year. Patient had history of perinatal infection. On local examination of right upper limb radius was grossly small and deformed in lower one third position. Ulnar head was grossly deformed and prominent, movements were restricted with dorsiflexion and planter flexion of about each 0-10 degrees. The patient was managed with osteotomy of radius and ulna. Proximal ulna was aligned with distal radius with K wire and bone grafting at the osteotomized site. Patient was discharged on dynamic cock-up splint for finger range of movement exercises for 3 weeks and follow up advised sequentially.

**Figure 1:**
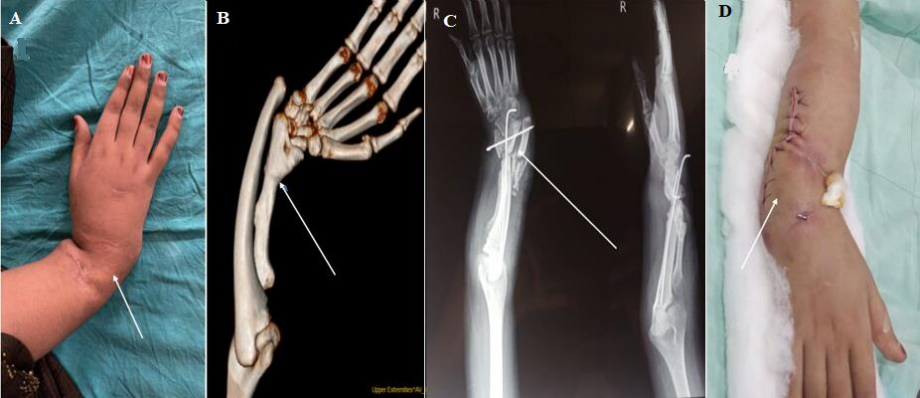
A) gross deformity of right forearm with prominent ulnar head; B) 3D-computed tomography image showing deformed ulna with radio ulnar dislocation with short radius; C) post operative X-ray showing fixation of deformity by k wire in situ; D) correction of forearm angulation deformity

